# Factors that Affect Intravenous Patient-Controlled Analgesia for Postoperative Pain Following Orthognathic Surgery for Mandibular Prognathism

**DOI:** 10.1371/journal.pone.0098548

**Published:** 2014-06-03

**Authors:** Yoshinori Aoki, Kaori Yoshida, Daisuke Nishizawa, Shinya Kasai, Tatsuya Ichinohe, Kazutaka Ikeda, Ken-ichi Fukuda

**Affiliations:** 1 Addictive Substance Project, Tokyo Metropolitan Institute of Medical Science, Tokyo, Japan; 2 Department of Dental Anesthesiology, Tokyo Dental College, Tokyo, Japan; United Graduate School of Child Development, Osaka University, Japan

## Abstract

The predictors of postoperative pain and analgesic consumption were previously found to include preoperative pain, anxiety, age, type of surgery, and genotype, but remaining unclear was whether intraoperative factors could predict postoperative pain. In the present study, we investigated the time-course of fentanyl consumption using intravenous patient-controlled analgesia records from patients who underwent orthognathic surgery for mandibular prognathism and analyzed the influence of anesthesia methods and surgical methods together with sex on the time course. A significant difference in the time course of fentanyl administration was found (*P*<0.001). No significant difference in the time course of fentanyl administration was found between males and females (*P* = 0.653), with no interaction between time course and sex (*P* = 0.567). No significant difference in the time course of fentanyl administration was found among anesthesia methods, such as fentanyl induction followed by fentanyl maintenance, fentanyl induction followed by remifentanil maintenance, and remifentanil induction followed by remifentanil maintenance (*P* = 0.512), but an interaction between time course and anesthesia method was observed (*P* = 0.004). A significant difference in the time course of fentanyl administration was found between surgical methods, such as bilateral mandibular sagittal split ramus osteotomy (BSSRO) and BSSRO combined with Le Fort I osteotomy (bimaxillary; *P* = 0.008), with no interaction between time course and surgical method (*P* = 0.535). Total postoperative 24 h consumption associated with the bimaxillary procedure was significantly higher than with BSSRO (*P* = 0.008). The present results indicate that administration patterns and total 24 h consumption were different among the three groups of anesthesia methods and between the two groups of surgical methods, respectively. Although more research on patient-controlled analgesia patterns and consumption is necessary, the present study will contribute to adequately relieving individual patients from postoperative pain.

## Introduction

Every year, 234.2 million major surgical procedures are performed worldwide [1]. These patients experience postoperative pain, and the range of pain varies from mild to severe. Postoperative pain management is very important to reduce distress caused by pain itself, contribute to cardiovascular stability [2] and proper respiratory function [3], and enable early recovery [3]. Postoperative pain is frequently controlled by opioids, which are especially heavily used in the United States [4]. Postoperative pain is reportedly affected by preoperative pain, anxiety, age, and type of surgery, and postoperative analgesic consumption is affected by type of surgery, age, and psychological distress [5]. Clarification and the control of preoperative and intraoperative factors will provide patients with more effective pain management.

In the present study, we investigated the time course of fentanyl consumption using the intravenous patient-controlled analgesia (IV-PCA) records of patients who underwent orthognathic surgery for mandibular prognathism and analyzed the factors (e.g., sex, anesthesia method, and surgical method) that may influence postoperative pain management. We found that the time course of IV-PCA was associated with the anesthesia method (i.e., time course × anesthesia method interaction) and surgical method (i.e., main effect).

## Materials and Methods

### 1. Patients

The study protocol was approved by the Institutional Review Boards of Tokyo Dental College and the Tokyo Metropolitan Institute of Medical Science. Written informed consent was obtained from all of the patients and from parents if the patient was under 20 years old. Enrolled in the study were 143 healthy patients (American Society of Anesthesiologists Physical Status I [ASA PS I], 15–53 years old, 56 males and 87 females) who were scheduled to undergo orthognathic surgery for mandibular prognathism at Tokyo Dental College Suidobashi Hospital. Patients were excluded preoperatively if they had a history of acute or chronic kidney injury, drug abuse, or chronic pain or were unable to use the IV-PCA device.

### 2. Anesthesia

The groups comprised consecutive patients who underwent cosmetic orthognathic surgery for mandibular prognathism and received fentanyl induction and maintenance (F-F group) over a half-year period prior to 2010, consecutive patients who underwent the same surgery and received fentanyl induction and remifentanil maintenance (F-R group) in the first half of 2010, and consecutive patients who underwent the same surgery and received remifentanil induction and maintenance (R-R group) in the second half of the year 2010. All of the groups were orally premedicated with 5 mg diazepam and 150 mg famotidine 90 min before the induction of anesthesia.

In the F-F group, the patients were inducted with 2 µg/kg fentanyl. General anesthesia was performed with propofol at a target blood concentration of 4–6 µg/ml using a target-controlled infusion (TCI) pump (TE-317, Terumo, Tokyo, Japan). Vecuronium (0.1 mg/kg) was administered to facilitate nasotracheal intubation (Portex; inner diameter, 6.5–8.0 mm; Smiths Medical Japan, Tokyo, Japan) and maintained at 0.08 mg/kg/h during surgery. Whenever systolic blood pressure or heart rate increased more than 20% over baseline during surgery, fentanyl was intravenously administered at 1 µg/kg.

In the F-R group, the patients were inducted with 2 µg/kg fentanyl. General anesthesia was performed with propofol at a target blood concentration of 4–6 µg/ml using a TCI pump. Recuronium (0.6 mg/kg) was administered to facilitate nasotracheal intubation (Portex; inner diameter, 6.5–8.0 mm; Smiths Medical Japan, Tokyo, Japan) (Portex; inner diameter, 6.5–8.0 mm; Smiths Medical Japan, Tokyo, Japan). General anesthesia was maintained with 0.125–0.5 µg/kg/min remifentanil and 7 µg/kg/min recuronium during surgery. The patients received 100 µg fentanyl as a transitional opioid at the end of surgery.

In the R-R group, the patients were inducted with 0.5 µg/kg/min remifentanil. General anesthesia was performed with propofol at a target blood concentration of 4–6 µg/ml using a TCI pump. Recuronium (0.6 mg/kg) was administered to facilitate nasotracheal intubation (Portex; inner diameter, 6.5–8.0 mm; Smiths Medical Japan, Tokyo, Japan). General anesthesia was maintained with 0.125–0.5 µg/kg/min remifentanil and 7 µg/kg/min recuronium during surgery. The patients received 100 µg fentanyl as a transitional opioid at the end of surgery.

In the three groups, the lungs were ventilated with oxygen-enriched air. All of the patients received local anesthesia at the surgical sites with 8 ml of 2% lidocaine that contained 12.5 µg/ml epinephrine.

### 3. Surgery

Sagittal split osteotomy described by Obwegeser [6] is likely the most frequently used procedure for osteotomy to correct mandibular anomalies, including hypoplasia, hyperplasia, and asymmetries. Le Fort I osteotomy, first described by Wassmund [7] and later standardized by Obwegeser [8] and Bell [9], has also become the most frequently used procedure for osteotomy in the maxilla [10]. In the present study, the surgical methods for mandibular prognathism included bilateral mandibular sagittal split ramus osteotomy (BSSRO) and BSSRO combined with Le Fort I osteotomy (bimaxillary). Bimaxillary surgery was performed for patients who had been deemed to present only marginal improvements in mandibular prognathism after BSSRO alone.

### 4. Postoperative pain management

At the end of surgery, 50 mg rectal diclofenac sodium and 8 mg intravenous dexamethasone were administered to prevent postoperative orofacial edema/swelling. After emergence from anesthesia and tracheal extubation, 1.25 mg droperidol was intravenously administered to prevent nausea/vomiting, and IV-PCA with 20 µg/ml fentanyl commenced using a CADD-Legacy PCA pump (Smiths Medical Japan, Tokyo, Japan). Droperidol (0.1 mg/ml) was co-administered with fentanyl to prevent nausea/vomiting because of a high incidence (up to 30%) of nausea/vomiting with PCA fentanyl in young females [11]. A bolus dose of fentanyl of 20 µg on demand and a lockout time of 10 min were set. Continuous background infusion was not employed. Patient-controlled analgesia was continued for 24 h postoperatively. In the case of refractory adverse effects or inadequate analgesia, PCA with fentanyl was discontinued, and 50 mg rectal diclofenac sodium was prescribed as a rescue analgesic as required.

The PCA pump recorded all of the administration events, providing the researchers with the administration times, number of administrations, dose of the administrations, and number of attempts without administration. The number of administrations was converted to consumption every 2 h after the end of anesthesia. Consumption every 2 h was standardized by body weight. Total postoperative 24 h consumption was calculated as the sum of consumption every 2 h. In 63 of the 143 cases, the intensity of spontaneous pain was assessed 3 and 24 h postoperatively using a 100 mm visual analog scale (VAS), with 0 mm indicating no pain and 100 mm indicating the worst pain imaginable.

### 5. Statistical analysis

All of the data are expressed as mean ± SD or median (range) and were statistically analyzed using SPSS 19.0 software (SPSS, Chicago, IL, USA). Differences between groups and within time courses were assessed using mixed-design analysis of variance (ANOVA; one-way for independent groups and repeated measures with Huynh-Feldt correction). When a significant overall effect was detected, Bonferroni's test and Scheffe's test were used to compare the mean values of the groups and time courses, respectively. Differences between groups in total postoperative 24 h consumption were analyzed using one-way ANOVA. The threshold for statistical significance was *P*<0.05. The sample size for the present data was higher than the estimated size that possesses statistical power (1 minus type II error probability) of 98% for the Cohen's conventional “medium” effect size of 0.3. Power analyses were performed using G*Power v.3.1.5 [12].

## Results

The attributes of the patients are shown in Table 1.

**Table 1:** Number of patients, age, sex, anesthesia methods, and surgical methods.

Sex	*n*, Age: median (range)	Anesthesia	*n*, Age: median (range)	Surgery	*n*, Age: median (range)
Male	56, 22.5 (16–53) years	F-F	44, 25.5 (16–53) years	BSSRO	94, 23.0 (15–49) years
		F-R	40, 22.0 (15–47) years		
Female	87, 25.0 (15–50) years			Bimaxillary	49, 25.0 (16–53) years
		R-R	59, 25.0 (16–50) years		
Total	143, 25.0 (15–53) years	Total	143, 25.0 (15–53) years	Total	143, 25.0 (15–53) years

### 1. Sex

**Figure 1:**
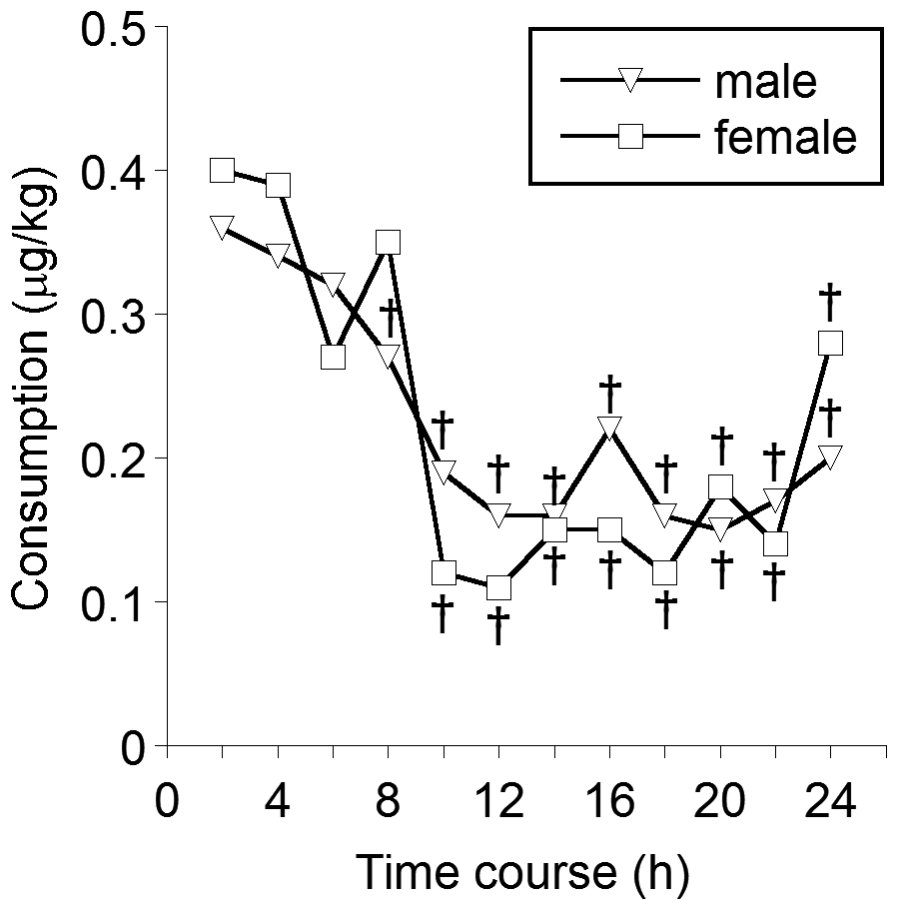
Differences in the time course of fentanyl administration between males and females. Main effects, interactions, and differences within time courses were analyzed using mixed-design ANOVA (one-way for independent groups and repeated-measures with Huynh-Feldt correction). The values indicate the medians. ^†^ *P*<0.05, compared with fentanyl consumption in the first 2 h.

A significant difference was found in the time course of fentanyl administration (*F*_6.435,907.356_ = 24.211, *MSe* = 0.166, *P*<0.001, Huynh-Feldt). In the time course, 2 h consumption significantly decreased from 6 h to 24 h after the end of anesthesia compared with consumption in the first 2 h (Fig. 1). No significant difference was found in fentanyl administration between males and females (*F*_1,141_ = 0.204, *MSe* = 0.593, *P* = 0.653, Huynh-Feldt), with no time course × sex interaction (*F*_6.435,907.356_ = 0.814, *MSe* = 0.166, *P* = 0.567, Huynh-Feldt). No significant difference was found in total postoperative 24 h consumption between males and females (Table 2; *F*_1,141_ = 0.204, *MSe* = 7.115, *P* = 0.653).

**Table 2:** Total postoperative 24

Subjects	*n*	Total 24 h consumption (µg/kg)	
		median	range
Sex			
Male	56	2.88	0.00–10.00
Female	87	2.40	0.00–11.34
Total	143	2.59	0.00–11.34
Anesthesia method			
F-F	44	2.64	0.00–10.54
F-R	40	2.28	0.00–9.07
R-R	59	2.70	0.00–11.34
Total	143	2.59	0.00–11.34
Surgical method			
BSSRO	94	2.29	0.00–9.07
Bimaxillary	49	3.16*	0.00–11.34
Total	143	2.59	0.00–11.34

*p<0.05, significant difference between BSSRO and bimaxillary groups.

### 2. Anesthesia methods

**Figure 2:**
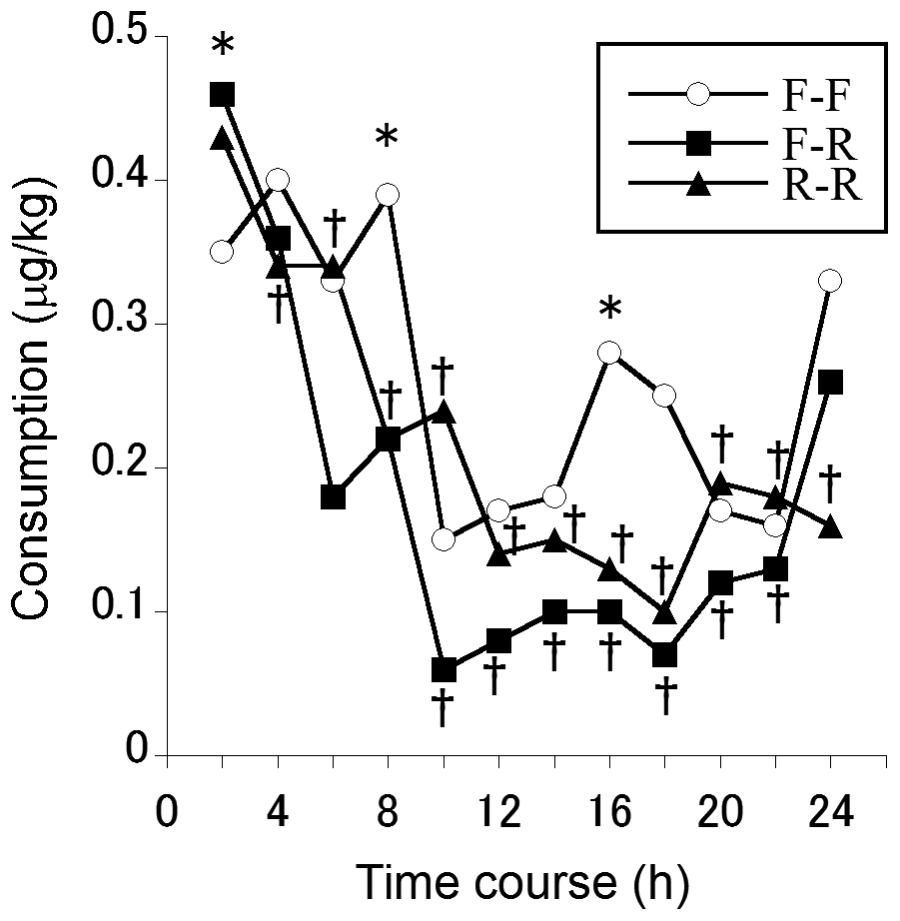
Differences in the time course of fentanyl administration among anesthesia methods. Main effects, interactions, and differences within time courses were analyzed using mixed-design ANOVA (one-way for independent groups and repeated-measures with Huynh-Feldt correction). F-F, fentanyl induction followed by fentanyl maintenance; F-R, fentanyl induction followed by remifentanil maintenance; R-R, remifentanil induction followed by remifentanil maintenance. The values indicate the medians. ^†^ *P*<0.05, compared with fentanyl consumption in the first 2 h; *P<0.05, significant difference among the three groups in 2 h fentanyl consumption.

A significant difference was found in the time course of fentanyl administration (*F*_6.661,932.489_ = 24.653, *MSe* = 0.157, *P*<0.001, Huynh-Feldt). In the time course, 2 h consumption significantly decreased from 6 h to 24 h after the end of anesthesia compared with consumption in the first 2 h (Fig. 2). No significant difference was found in fentanyl administration among the anesthesia methods (*F*_2,140_ = 0.672, *MSe* = 0.592, *P* = 0.512, Huynh-Feldt), but a significant time course × anesthesia method interaction was observed (*F*_13.321,932.489_ = 2.359, *MSe* = 0.157, *P* = 0.004, Huynh-Feldt). Consumption in the first 2 h in the R-R group was significantly higher than in the F-F group, but 8 h consumption in the R-R and F-R groups was significantly lower than in the F-F group. Nevertheless, total postoperative 24 h consumption was not significantly different among the three groups (Table 2; *F*_2,141_ = 0.672, *MSe* = 7.108, *P* = 0.512).

### 3. Surgical methods

**Figure 3:**
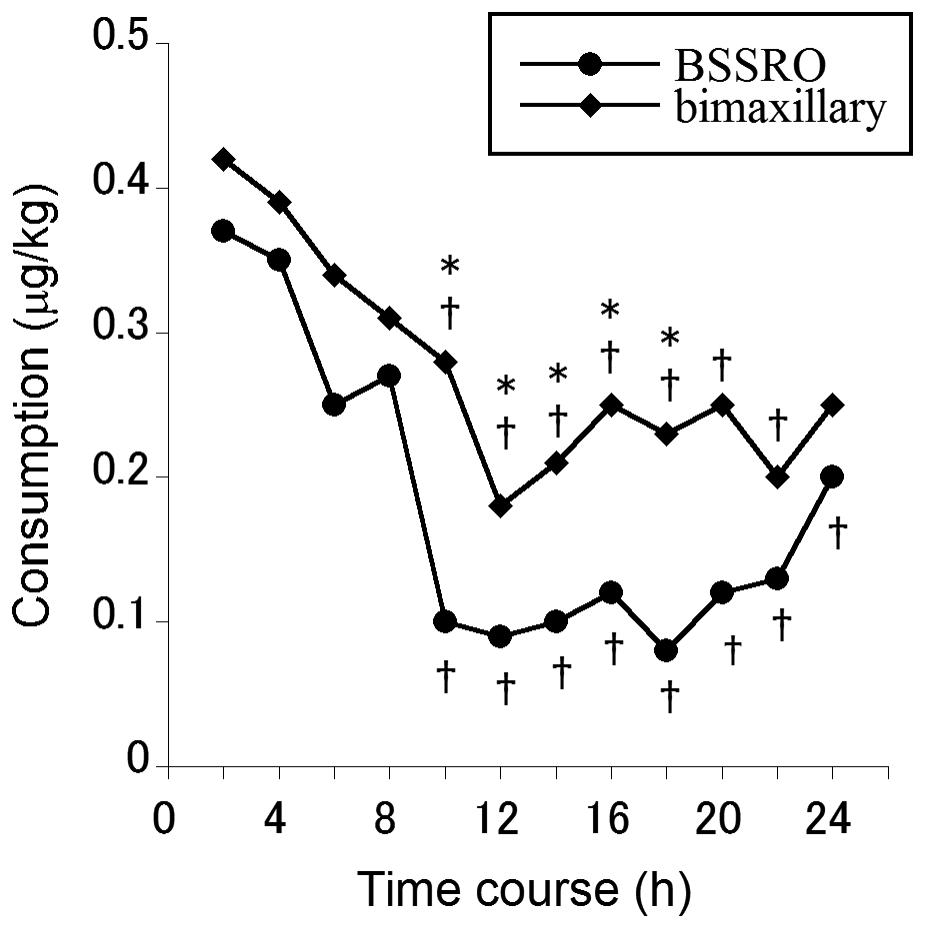
Differences in the time course of fentanyl administration between surgical methods. Main effects, interactions, and differences within time courses were analyzed using mixed-design ANOVA (one-way for independent groups and repeated-measures with Huynh-Feldt correction). The values indicate the medians. ^†^ *P*<0.05, compared with fentanyl consumption in the first 2 h; *P<0.05, significant difference between the BSSRO and bimaxillary groups.

A significant difference was found in the time course of fentanyl administration (*F*_6.482,913.936_ = 24.144, *MSe* = 0.165, *P*<0.001, Huynh-Feldt). In the time course, 2 h consumption significantly decreased from 4 h to 24 h after the end of anesthesia compared with consumption in the first 2 h. A significant difference was found in fentanyl administration between the surgical methods (*F*_1,141_ = 7.237, *MSe* = 0.565, *P* = 0.008, Huynh-Feldt), but no time course × surgical method interaction was observed (*F*_6.482,913.936_ = 0.855, *MSe* = 0.165, *P* = 0.535, Huynh-Feldt). Consumption in the first 2 h was higher in the bimaxillary group than in the BSSRO group (Fig. 3). Total postoperative 24 h consumption in the bimaxillary group was significantly higher than in the BSSRO group (Table 2; *F*_1,141_ = 7.237, *MSe* = 6.778, *P* = 0.008, Huynh-Feldt).

### 4. Visual analog scale

The attributes of the patients are shown in Table S1. No significant difference was found in VAS scores between the anesthesia methods (F-F and F-R groups) at 3 h (*t*_61_ = −0.713, *P* = 0.478) and 24 h (*t*_61_ = −0.098, *P* = 0.992). A significant positive correlation was found between total postoperative 24 h consumption and VAS scores at 3 h, but the correlation coefficient was relatively small (*r* = 0.295, *P* = 0.019). No significant positive correlation was found between total postoperative 24 h consumption and VAS scores at 24 h (*r* = 0.240, *P* = 0.058). A significant positive correlation was found between VAS scores at 3 and 24 h, and the correlation coefficient was relatively large (*r* = 0.667, *P*<0.001).

## Discussion

The predictors of postoperative pain were previously found to include preoperative pain, anxiety, age, type of surgery [5], and genotype [11], [13]–[15]. We investigated orthognathic patients in whom these predictive factors are considered to be relatively similar. They had been treated by a few orthodontists in the hospital over several years. Their anxiety appeared to be much less than patients who presented in the emergency room. Almost all of the patients were young (mean age = 23.16 years, SD = 0.696 years) and healthy (ASA PS I). Orthognathic surgery was performed after body growth ceased. Orthognathic procedures, such as BSSRO and BSSRO combined with Le Fort I osteotomy, have been well established. The patients were subjected to uniform invasiveness by these typical operations. Postoperative pain after BSSRO has been reported to be more intense than after soft tissue surgery [16]. Thus, these patients had no preoperative pain (e.g., inflammatory pain), had less anxiety, were young, and had similar levels postoperative pain; therefore, they were deemed to be suitable for inclusion as subjects to investigate the factors that influence the time course of IV-PCA.

Young patients were reported to be more sensitive to postoperative pain than older patients [5]. We did not analyze the association between age and postoperative fentanyl consumption because our data were collected mainly from young patients.

Generally, sex is not associated with postoperative pain [5], although postoperative pain in patients who underwent impacted third molar extraction was associated with sex [17]. Pain assessed by a visual analog scale (VAS) during the first 24 h in females was significantly higher than in males, but the VAS score after the first 24 h was not significantly different between males and females [17]. The initially higher level of pain in females may be attributable to a smaller and thinner mandible in females [17]. The surgical site in the present study was similar to impacted third molar extraction; thus, we analyzed the association between sex and postoperative fentanyl consumption. However, our data did not show a significant difference in fentanyl consumption between males and females. Impacted third molar extraction might cause more micro-bone fractures in females than in males. Because osteotomy is not a micro-bone fracture but rather an artificial fracture, postoperative fentanyl consumption might not have been affected by sex differences in the structure of the mandible in the present study.

Anesthesia methods were analyzed by two-way ANOVA without sex as a covariate because no significant difference was found between males and females in the present study. A time course × anesthesia method interaction was observed, in which consumption in the first 2 h was higher than 4 h consumption in the R-R and F-R groups, but 2 h consumption was lower than 4 h consumption in the F-F group. The context-sensitive half-life of remifentanil is extremely less than fentanyl [18]. The recovery of psychomotor function after total intravenous anesthesia (TIVA) with remifentanil, which does not use any inhalational agents, was 30–120 min faster than TIVA with fentanyl [19]. Orthognathic patients who were maintained with TIVA with remifentanil had significantly higher pain scores within the first 4 h postoperatively [20]. Thus, the groups that were maintained with remifentanil (i.e., the F-R and R-R groups) may recognize postoperative pain father than the group that was maintained with fentanyl (i.e., the F-F group). Faster psychomotor recovery and the faster recognition of pain might explain why the groups that were maintained with remifentanil had higher fentanyl consumption in the first 2 h than the group that was maintained with fentanyl. Interestingly, the administration pattern was different between the remifentanil and fentanyl groups, but total postoperative 24 h consumption was not different among the three groups of anesthesia methods. Additionally, the 3 and 24 h VAS scores were mostly less than 50 mm and not different between anesthesia methods (F-F and F-R groups), indicating that subjective pain was appropriately controlled in both the F-F and F-R groups.

We had empirically known that bimaxillary surgery is experimentally more painful than BSSRO. Postoperative pain following BSSRO and Le Fort I osteotomy is conveyed from the surgical sites to supraspinal sites by the third and second branches of the trigeminal nerve, respectively. Thus, postoperative pain following bimaxillary surgery was conveyed from the surgical sites to supraspinal sites by both the second and third branches of the trigeminal nerve. Our results suggest that postoperative pain increased because of the increase in the number of branches of the trigeminal nerve from the surgical site. Further studies of single Le Fort I osteotomy (second branch of the trigeminal nerve) are required to determine whether the increase in postoperative pain is caused by synergistic or additive effects.

The present study involved healthy patients who underwent oral surgery. The influence of perioperative factors on IV-PCA was controlled. The results showed that the administration patterns and total 24 h consumption were different among the three groups of anesthesia methods and between the two groups of surgical methods, respectively. Although more research on patient-controlled analgesia patterns and consumption is necessary, the present study will contribute to adequately relieving individual patients from postoperative pain.

## Supporting Information

Table S1 **Frequency of patients and VAS scores.**
(DOCX)

## References

[1] Weiser TG, Regenbogen SE, Thompson KD, Haynes AB, Lipsitz SR, et al (2008) An estimation of the global volume of surgery: a modelling strategy based on available data. Lancet 372: 139–144 10.1016/S0140-6736(08)60878-8.PMID:18582931 18582931

[2] Warltier DC, Pagel PS, Kersten JR (2000) Approaches to the prevention of perioperative myocardial ischemia. Anesthesiology 92: 253–259. PMID: 10638923. 10.1097/00000542-200001000-00038 10638923

[3] Kehlet H, Holte K (2002) Effect of postoperative analgesia on surgical outcome. Br J Anaesth 87: 62–72. PMID: 12024074. 10.1093/bja/87.1.62 11460814

[4] United Nations (2011) Report of the International Narcotics Control Board for the availability of internationally controlled drugs: ensuring adequate access for medical and scientific purposes. New York: United Nations. pp. 59.

[5] Ip HY, Abrishami A, Peng PW, Wong J, Chung F (2009) Predictors of postoperative pain and analgesic consumption: a qualitative systematic review. Anesthesiology 111: 657–677 10.1097/ALN.0b013e3181aae87a.PMID:19672167 19672167

[6] Trauner R, Obweser H (1957) The surgical correction of mandibular prognathism and retrognathia with consideration of genioplasty. I. Surgical procedures to correct mandibular prognathism and reshaping of the chin. Oral Surg Oral Med Oral Pathol 10: 677–689. PMID: 13441284. 10.1016/s0030-4220(57)80063-2 13441284

[7] Wassmund M (1935) Lehrbuch der Praktischen Chirurgie des Mundes und der Kiefer, Bd I. Berlin: Meusser. pp. 282–284.

[8] Obweser H (1965) Surgery of the maxilla for the correction of prognathism. SSO Schweiz Monatsschr Zahnheilkd 75: 365–374. PMID: 14280763. 14280763

[9] Bell WH (1975) Le Forte I osteotomy for correction of maxillary deformities. J Oral Surg 33: 412–426. PMID: 1055202. 1055202

[10] Haerle F, Champy M, Terry B (1997) Atlas of Craniomaxillofacial Osteosynthesis: Microplates, miniplates, and screws. Stuttgart: Thieme Medical Publishers. pp. 90, 100.

[11] Fukuda K, Hayashida M, Ide S, Saita N, Kokita Y, et al (2009) Association between *OPRM1* gene polymorphisms and fentanyl sensitivity in patients undergoing painful cosmetic surgery. Pain 147: 194–201 10.1016/j.pain.2009.09.004.PMID:19783098 19783098

[12] Faul F, Erdfelder E, Lang AG, Buchner A (2007) G*Power 3: a flexible statistical power analysis program for the social, behavioral, and biomedical sciences. Behav Res Methods 39: 175–191. PMID: 17695343. 10.3758/bf03193146 17695343

[13] Nishizawa D, Nagashima M, Katoh R, Satoh Y, Tagami M, et al (2009) Association between *KCNJ6* (*GIRK2*) gene polymorphisms and postoperative analgesic requirements after major abdominal surgery. PLoS One 4: e7060 10.1371/journal.pone.0007060.PMID:19756153 19756153 PMC2738941

[14] Nishizawa D, Fukuda K, Kasai S, Hasegawa J, Aoki Y, et al (2014) Genome-wide association study identifies a potent locus associated with human opioid sensitivity. Mol Psychiatry 19: 55–62 10.1038/mp.2012.164.PMID:23183491 23183491 PMC3873034

[15] Aoki Y, Nishizawa D, Kasai S, Fukuda K, Ichinohe T, et al (2013) Association between the variable number of tandem repeat polymorphism in the third exon of the dopamine D4 receptor gene and sensitivity to analgesics and pain in patients undergoing painful cosmetic surgery. Neurosci Lett 542: 1–4 10.1016/j.neulet.2013.02.039.PMID:23458670 23458670

[16] Nagatsuka C, Ichinohe T, Kaneko Y (2000) Preemptive effects of a combination of preoperative diclofenac, butorphanol, and lidocaine on postoperative pain management following orthognathic surgery. Anesth Prog 47: 119–124. PMID: 11432176. PMC2149035 11432176

[17] de Santana-Santos T, de Souza-Santos aA, Martins-Filho PR, da Silva LC, de Oliveira E Silva ED, et al. (2013) Prediction of postoperative facial swelling, pain and trismus following third molar surgery based on preoperative variables. Med Oral Patol Oral Cir Bucal 18: e65–e70. PMID: 23229245. 10.4317/medoral.18039 PMC3548647 23229245

[18] Bürkle H, Dunbar S, Van Aken H (1996) Remifentanil: a novel, short-acting, mu-opioid. Anesth Analg. 83: 646–651. PMID: 8780298. 10.1097/00000539-199609000-00038 8780298

[19] Takayama A, Yamaguchi S, Ishikawa K, Shinozaki M, Kimura Y, et al (2012) Recovery of psychomotor function after total intravenous anesthesia with remifentanil-propofol or fentanyl-propofol. 26: 34–38 10.1007/s00540-011-1266-5.PMID:22048284 22048284

[20] Chegini S, Johnston KD, Kalantzis A, Dhariwal DK (2012) The effect of anesthetic technique on recovery after orthognathic surgery: a retrospective audit. Anesth Prog 59: 69–74 10.2344/11-10.1.PMID:22822993 22822993 PMC3403584

