# Supplementary material for: Factors that Affect Intravenous Patient-Controlled Analgesia for Postoperative Pain Following Orthognathic Surgery for Mandibular Prognathism
Source: PLoS One. 2014 Jun 3;9(6):e98548. doi: 10.1371/journal.pone.0098548 (PMC4043772; doi:10.1371/journal.pone.0098548)
Supplement: Table S1 — Frequency of patients and VAS scores. (DOCX) [file pone.0098548.s001.docx]

Table S1. Frequency of patients and VAS scores.

| Groups | | | *n* | VAS 3 h (mm) | | VAS 24 h (mm) | |
| --- | --- | --- | --- | --- | --- | --- | --- |
|  |  |  |  | mean | ± SD | mean | ± SD |
| Sex | | |  |  |  |  |  |
|  | | Male | 22 | 37.2 | ± 23.54 | 28.1 | ± 22.59 |
|  | | Female | 41 | 41.3 | ± 21.27 | 30.4 | ± 21.08 |
| Anesthesia method | | |  |  |  |  |  |
|  | F-F | | 26 | 37.5 | ± 21.34 | 29.9 | ± 20.34 |
|  | F-R | | 37 | 41.6 | ± 22.57 | 29.4 | ± 22.50 |
|  | R-R | | 0 |  |  |  |  |
| Surgical method | | |  |  |  |  |  |
|  | BSSRO | | 63 | 39.0 | ± 21.99 | 29.6 | ± 21.46 |
|  | Bimaxillary | | 0 |  |  |  |  |
